# The Use of Questionable Research Practices to Survive in Academia Examined With Expert Elicitation, Prior-Data Conflicts, Bayes Factors for Replication Effects, and the Bayes Truth Serum

**DOI:** 10.3389/fpsyg.2021.621547

**Published:** 2021-11-29

**Authors:** Rens van de Schoot, Sonja D. Winter, Elian Griffioen, Stephan Grimmelikhuijsen, Ingrid Arts, Duco Veen, Elizabeth M. Grandfield, Lars G. Tummers

**Affiliations:** ^1^Department of Methods and Statistics, Utrecht University, Utrecht, Netherlands; ^2^Optentia Research Program, Faculty of Humanities, North-West University, Vanderbijlpark, South Africa; ^3^Missouri Prevention Science Institute, University of Missouri, Columbia, MO, United States; ^4^Department of Global Health, Julius Center for Health Sciences and Primary Care, University Medical Center Utrecht, Utrecht, Netherlands; ^5^School of Governance, Utrecht University, Utrecht, Netherlands

**Keywords:** informative prior, Bayes truth serum, expert elicitation, replication study, questionable research practices, Ph.D. students, Bayes Factor (BF)

## Abstract

The popularity and use of Bayesian methods have increased across many research domains. The current article demonstrates how some less familiar Bayesian methods can be used. Specifically, we applied expert elicitation, testing for prior-data conflicts, the Bayesian Truth Serum, and testing for replication effects via Bayes Factors in a series of four studies investigating the use of questionable research practices (QRPs). Scientifically fraudulent or unethical research practices have caused quite a stir in academia and beyond. Improving science starts with educating Ph.D. candidates: the scholars of tomorrow. In four studies concerning 765 Ph.D. candidates, we investigate whether Ph.D. candidates can differentiate between ethical and unethical or even fraudulent research practices. We probed the Ph.D.s’ willingness to publish research from such practices and tested whether this is influenced by (un)ethical behavior pressure from supervisors or peers. Furthermore, 36 academic leaders (deans, vice-deans, and heads of research) were interviewed and asked to predict what Ph.D.s would answer for different vignettes. Our study shows, and replicates, that some Ph.D. candidates are willing to publish results deriving from even blatant fraudulent behavior–data fabrication. Additionally, some academic leaders underestimated this behavior, which is alarming. Academic leaders have to keep in mind that Ph.D. candidates can be under more pressure than they realize and might be susceptible to using QRPs. As an inspiring example and to encourage others to make their Bayesian work reproducible, we published data, annotated scripts, and detailed output on the Open Science Framework (OSF).

## Introduction

Several systematic reviews have shown that applied researchers have become more familiar with the typical tools of the Bayesian toolbelt (Johnson et al., 2010a; König and van de Schoot, 2017; van de Schoot et al., 2017, 2021a; Fragoso et al., 2018; Smid et al., 2020; Hon et al., 2021). However, there remain many tools in the Bayesian toolbelt that are less familiar in the applied literature. In the current article, we illustrate how some less familiar tools can be applied to empirical data: A Bayesian expert-elicitation method (O’Hagan et al., 2006; Anca et al., 2021) – also described in van de Schoot et al. (2021b), a test for prior-data conflict using the prior predictive *p*-value (Box, 1980) and the Data Agreement Criterion (DAC) (Veen et al., 2018), a Bayes truth serum to correct for socially desirable responses (Prelec, 2004), and testing for replication effects via the Bayes Factor (Bayarri and Mayoral, 2002; Verhagen and Wagenmakers, 2014). These methods are applied to the case of how Ph.D. students respond to academic publication pressure in terms of conducting questionable research practices (QRPs).

In what follows, we first elaborate on QRPs, how Ph.D. candidates respond to scenarios of QRPs, and senior academic leaders (deans, heads of departments, and research directors, etc.) believe Ph.D. candidates will deal with this pressure. In four separate sections, we present the results of the different studies and illustrate how the Bayesian methods mentioned above can be applied to answer the substantive research questions, thereby providing an example of how to use Bayesian methods for empirical data. Also, Supplementary Material, including annotated code, part of the anonymized data, and more detailed output files, can be found on the Open Science Framework (OSF)^1^. The Ethics Committee of the Faculty of Social and Behavioral Sciences at Utrecht University approved the series of studies (FETC15-108), and the questionnaires were co-developed and pilot-tested by a university-wide organization of Ph.D. candidates at Utrecht University (Prout) and the Dutch National Organization of Ph.D. candidates (PNN). Supplementary Appendix A–C contains additional details referred to throughout the text.

## The Case of Questionable Research Practices to Survive in Academia

Science has always been a dynamic process with continuously developing and often implicit rules and attitudes. While a focus on innovation and knowledge production are essential to academic progress, it is equally important to convey and stimulate the use of the most appropriate research practices within the academic community (Martinson et al., 2005; Fanelli, 2009; Tijdink et al., 2014). There is an intense pressure to publish since scientific publications are integral in obtaining grants or obtaining a tenured position in academia (Gopalakrishna et al., 2021; Haven et al., 2021). Ph.D. candidates have noted that the most critical factors related to obtaining an academic position were the number of papers presented, submitted, and accepted in peer-reviewed journals (Sonneveld et al., 2010; Yerkes et al., 2010). In an observational study by Tijdink et al. (2014), 72% of respondents reported pressure to publish was “too high” and was associated with higher scores on a scientific misconduct questionnaire measuring self-reported fraud and QRPs. With increasing publication pressure, a growing number of scholars, and ever more interdisciplinary and international studies being conducted, academic norms have become diverse and complicated. Publication pressure combined with the ambiguity of academic standards has contributed to QRPs such as data fabrication, falsification, or other modifications of research results (Fanelli, 2010). Early-career scientists may struggle to identify QRPs and, as Sijtsma (2016) noted, may even commit QRPs unintentionally. Anecdotally, statements such as “this is how we always do it,” “get used to it,” or “this is what it takes to survive in academia” may also be familiar to some researchers and students, which do not help develop a sense of ethical standards for research practices.

In response to these observations, the contemporary debate about appropriate scientific practices is fierce and lively and has extended to non-academic domains. Therefore, how we conduct research and, equally important, how we inform, mentor, and educate young scientists is essential to sound scientific progress and how science is perceived and valued (Anderson et al., 2007; Kalichman and Plemmons, 2015). An observational study by Heitman et al. (2007), for example, found that scholars who reported receiving education about QRPs scored 10 points higher on a questionnaire about these issues (reporting that they are less likely to participate in QRPs) compared to scholars without prior QRP training. A Ph.D. trajectory is essentially about educating someone to become an independent scientist, ethical research practices should be part of all graduate curricula. Still, early-career scientists mostly learn from observing the scientific norms and practices of academic leaders (Hofmann et al., 2013), most of whom are their direct supervisors. Ph.D. candidates are in a highly dependent relation with these senior faculty members. Senior faculty, therefore, is in the position to influence the Ph.D. candidate, which also holds for ethical issues concerning scientific behavior. At the same time, Ph.D. candidates compete with their peers for a limited number of faculty positions, a situation that may also be a factor in yielding to questionable scientific behavior.

The various potential sources of pressure from senior academic leaders and peer competition occur in an early stage of their academic career when Ph.D. candidates are susceptible to learning about ethical research practices. Senior researchers with a role-model function may not completely understand the pressure experienced by the current cohort of Ph.D. candidates. It has, so far, never been investigated how such pressure interacts with the occurrence of questionable research behavior among Ph.D. candidates, nor how academic leaders predict the behavior of Ph.D. candidates in such situations.

Therefore, in the current article, we present a series of four studies investigating these issues.

For the first study, we asked Ph.D. candidates from a wide range of Social Sciences faculties across Netherlands what they would do when faced with the three scenarios, how they would respond, to whom to talk about it, and whether they had experienced a similar situation in their career. We also added experimental conditions: in the description of the senior scholar for the vignettes, we manipulated the level of ethical leadership (high/low) and research transparency (high/low). Ethical leadership and research transparency were used as a manipulation check to see if participants interpreted the vignettes correctly. These two factors were included because in the organizational sciences, ethical leadership is thought to be a way to improve employees’ ethical conduct (Brown and Treviño, 2006), and increased research transparency is offered as a solution to prevent fraud and misconduct in many fields of science (Parker et al., 2016).

For the second study, we interviewed academic leaders about what they expected. Ph.D. candidates would do in the scenarios from Study 1. The social sciences within Netherlands had a real wake-up call with the Stapel case (Callaway, 2011; Levelt et al., 2012; Markowitz and Hancock, 2014). Hopefully, this case would have created awareness, at least in academic leaders. The question is whether the academic leaders would think the Ph.D. candidates, who mostly started their projects after the news about Stapel had faded away, also changed their attitude toward scientific fraud and QRPs. Therefore, after obtaining the results from the academic leaders, we tested for expert-data (dis)agreement (Bousquet, 2008; Veen et al., 2018) between the academic leaders and the Ph.D. candidates to see if the academic leaders over-or underestimated the replies given by the Ph.D. candidates.

The third study concerned a conceptual replication of the first vignette in Study 1 (data fabrication). Replication is not only an essential aspect of scientific research but has also been recommended as a method to help combat QRPs (Sijtsma, 2016; Sijtsma et al., 2016; Waldman and Lilienfeld, 2016). Study 3 participants were from a major university in Netherlands not included in Study 1 and represented Psychology and medical sciences. We also added two new scenarios (gift authorship and omitting relevant information) and a second experimental condition in which we manipulated peer and senior pressure by including cues in the vignette about the (imaginary) prevalence of QRPs of fellow Ph.D. candidates and professors at a different, fictional, university. It was based on the assumption that obedience to authority–from superiors or peers–influences questionable behavior, as evidenced by the large body of literature on the theory of planned behavior (Ajzen, 1991) and more general work on subjective norms and peer pressure (Terry and Hogg, 1996).

Finally, in Study 4, we replicated the experiment of Study 1 in a new sample outside the Netherlands, namely, in three Social Sciences faculties in Belgium. Replication studies are not only an essential aspect of science; as mentioned above, they may also aid in uncovering and potentially reducing QRPs.

## Study 1–Vignette Study A

There were two goals for Study 1: First, to investigate how Ph.D. candidates would respond to the vignettes about data fabrication, deleting outliers to get significant results, and salami slicing; see Supplementary Appendix A for the text used in the vignettes. Second, we used a randomized experiment to investigate whether characteristics in the description of the senior, in terms of ethical leadership and transparency, would influence their responses.

### Methods

#### Participants, Procedure, and Design

The Ph.D. candidates for Study 1 were recruited from 10 Social Sciences or Psychology faculties at eight universities in Netherlands out of 10 universities with Social Sciences or Psychology faculties. Two more universities were invited, but one declined to participate, and at the other, the data collection never got started due to practical issues. We always asked a third party (usually a Ph.D. organization within the university) to send invitations to their Ph.D. candidates to participate in our study. This procedure ensured that we were never in possession of the email addresses of potential participants. We used the online survey application, LimeSurvey, to create a separate, individualized survey for each university involved. To further ensure our participants’ privacy, we configured the surveys to save anonymized responses without information about IP address, the date and time they completed the survey, or the location of their computer (city and country). Furthermore, we ensured that all demographics questions were not mandatory for participants to complete to decide how much information they wished to share with us. Finally, participants were offered the possibility to leave an email address if they wanted to receive notice of the outcomes of our research. However, we never created a data file that contained both the email addresses and the survey data. Participants were randomly assigned to one of the four conditions within the survey.

In total, 440 Ph.D. candidates completed the questions for at least one scenario. Descriptive statistics about the sample can be found in Table 1. The survey focused on the three scenarios concerning QRPs/fraud: (1) data fabrication, (2) deleting outliers to get significant results, and (3) salami-slicing; see Supplementary Appendix A for the exact text we used. After presenting a scenario to the participant, we first asked an open-ended question: “What would you do in this situation?” Then we asked: “Would you (try to) publish the results coming from this research?” (Yes/No) followed by an open-ended question “If you want, you can elaborate on this below.”

**TABLE 1:** Descriptive statistics for Study 1 (*N* = 440), Study 3 (*N* = 198), and Study 4 (*N* = 127).

Variable		Study 1	Study 3	Study 4
Gender	Male	128 (29.09%)	48 (24.24%)	80 (62.99%)
	Female	247 (56.14%)	121 (61.11%)	35 (27.56%)
	Prefer not to disclose	12 (2.73%)	8 (4.04%)	2 (1.57%)
	Missing	53 (12.05%)	21 (10.61%)	10 (7.87%)
Age		31.65 (7.84; 24/70)	31.40 (6.15; 24/64)	29.06 (4.79; 23/48)
		*n* = 365	*n* = 166	*n* = 116
Employment type	Standard Ph.D. candidate	296 (67.27%)	150 (75.76%)	45 (35.43%)
	No Ph.D. candidate but Ph.D. scholarship	17 (3.86%)	10 (5.05%)	56 (44.09%)
	External Ph.D. candidate	15 (3.45%)	10 (5.10%)	7 (5.51%)
	Other	53 (12.01%)	20 (10.10%)	10 (7.87%)
	Missing	59 (13.41%)	8 (4.04%)	9 (7.09%)
Data: Collecting and/or analyzing	I collect and analyze	370 (84.09%)	139 (70.20%)	103 (81.10%)
	I collect, someone else analyses	20 (4.55%)	14 (7.07%)	4 (3.15%)
	I analyze existing data	37 (8.41%)	32 (16.16%)	14 (11.02%)
	My research is mainly theoretical	7 (1.59%)	9 (4.55%)	4 (3.15%)
	Missing	6 (1.36%)	4 (2.02%)	2 (1.57%)
Certainty career in academics	Scale 1–10	6.76 (2.27; 1/10)	6.82 (2.32; 1/10)	5.39 (2.56, 1/10)
		*n* = 440	*n* = 198	*n* = 127
Ambition career in academics	Scale 1–10	6.80 (2.20; 1/10)	6.91 (2.14; 1/10)	5.50 (2.49; 1/10)
		*n* = 440	*n* = 198	*n* = 127
Perceived publication pressure	Scale 1–6	4.64 (0.91; 1/6)		
Is publication pressure present in the research field?	Scale 1–10		7.11 (1.87; 1/10)	7.41 (1.77;1/10)

*Data are mean (SD; min/max) or frequency (%).*

We compared responses of the Ph.D. candidates across four conditions, which were combinations of two two-level factors, Leadership and Data. To convey these conditions to the participant, we used different combinations of the introductory texts. LimeSurvey allowed us to automatically and randomly assign participants to one of the four conditions for the first experiment and then again in one of the four conditions of the experiment.

To check whether participants perceived the manipulations (high versus low ethical leadership and high versus low research transparency), we included scales for both ethical leadership (Yukl et al., 2013) (Cronbach’s alpha 0.919) and research transparency (developed for this study, see Supplementary Appendix A for the questions used, Cronbach’s alpha 0.888). In Supplementary Appendix B, we describe the results of the manipulation checks for Ethical Leadership and Data Transparency. We concluded that the manipulation resulted in a different score on both variables across conditions, indicating that our manipulation was effective.

#### Analytic Strategy

We first provide descriptive statistics about the responses of the Ph.D. candidates to each of the vignettes.

Second, we present the replies to the open-ended questions. We grouped the responses in several categories. Grouping of the open answer was made based on group discussions and consensus among the authors using an *ad hoc* bottom-up process. Multiple categories could be given to each answer. We discussed ambiguous responses and only classified participants’ answers in one of the categories if all authors reached a consensus. We also examined whether, based on information in the open-ended questions, the Ph.D. candidates provided an honest reply to the yes/no question about publishing and recoded the item into a new variable next to the existing variable. For the first scenario, in 22 cases, the information in the open-ended answer did not correspond with the yes/no question. An equal number of responses was recoded from “yes” to “no” and from “no” to “yes.” For the second scenario, we recoded 154 answers. In most of these cases (97%), the Ph.D. candidate indicated in the open-ended answers that they would publish the results only if the outliers were described in the article. Since the scenario was about publishing the data without providing more information, we recoded these answers to “no.” As a result, the percentage of participants indicating that they would attempt to publish dropped from 48.8 to 12.5% (a 36.3% decline). In the third scenario, in 16 cases, the information in the open-ended answer did not correspond with the yes/no answer. It resulted in a decline of 1.5% in the participants’ indication that they would attempt to publish. Again, the decisions were discussed and only changed if consensus was reached among all authors.

Third, we used Bayes Factors for contingency tables in JASP (JASP-Team, 2018) to examine whether the experimental conditions affected the participants’ attitude toward publishing data or analyses that might have fallen victim to QRPs. When a hypothesis is tested against an alternative hypothesis, and the results indicate that BF ≈ 1 implies that both hypotheses are equally supported by the data. However, for example, when BF = 10, the support for one hypothesis is 10 times larger than the support for the alternative hypothesis. For interpretation of Bayes Factors, we refer interested readers to the classical paper of Kass and Raftery (1995).

### Results

Most Ph.D. candidates in this study (96.6%) answered “yes” to the question of whether they consider the vignette scenario to be fraudulent (see Table 2). As for the first scenario, almost all Ph.D. candidates believe data fabrication is fraudulent; interestingly, 5.9% (25 students) would still publish the results, and some participants reported having experienced such a situation.

**TABLE 2:** Results in percentages of the vignette studies Study 1 (*N* = 440), Study 3 (*N* = 198), and Study 4 (*N* = 127).

	Study 1			Study 3			Study 4		
	“Is this fraud?” (% Yes)	“Yes, I would try to publish”	“Have you experienced a similar situation?” (% Yes)	“Is this fraud?” (% Yes)	“Yes, I would try to publish”	“Have you experienced a similar situation?” (% Yes)	“Is this fraud?” (% Yes)	“Yes, I would try to publish”	“Have you experienced a similar situation?” (% Yes)
Scenario 1: Data fabrication*^a^*	96.6%	5.9%	3.2%	92.4%	9.6%	5.5%	92.9%	13.4%	5.5%
	(*n* = 440)	(*n* = 440)	(*n* = 440)	(*n* = 198)	(*n* = 198)	(*n* = 198)	(*n* = 127)	(*n* = 127)	(*n* = 127)
Scenario 2: Deleting outliers to get significant results	56.4%	12.3%	12.9%						
	(*n* = 407)	(*n* = 407)	(*n* = 407)						
Scenario 3: Salami slicing*^a^*	65.2%	32.0%	9.3%	16.6%	38.9%	17.2%	23.6%	32.8%	17.3%
	(*n* = 397)	(*n* = 397)	(*n* = 397)	(*n* = 185)	(*n* = 185)	(*n* = 185)	(*n* = 119)	(*n* = 119)	(*n* = 119)
Scenario 4: Gift authorship				42.4%	59.2%	30.3%	40.6%	58.8%	16.7%
				(*n* = 184)	(*n* = 184)	(*n* = 184)	(*n* = 118)	(*n* = 118)	(*n* = 116)
Scenario 5: Excluding information				71.7%	12.1%	13.6%	72.4%	16.1%	15.8%
				(*n* = 182)	(*n* = 182)	(*n* = 182)	(*n* = 118)	(*n* = 118)	(*n* = 118)

*^a^For Studies 3 and 4, we modified the description based on feedback from the participants, see Supplementary Appendix A.*

Most participants provided extensive answers to the open-ended questions. We grouped their responses into six categories. The first category comprised 34.6% of the Ph.D. candidates who indicated they would never publish such results because they feel *morally obliged* not to do so, as is implied by statements like “it wouldn’t feel good to do so” or “I can’t accept that for myself,” or put more strongly:

*“Never, this goes against all I stand for and this is not what research is about, I feel very annoyed that this question is even being asked.”*

The second category of Ph.D. candidates (22.6%) reported that they would first *talk to someone else before taking any action*. Of these Ph.D. candidates, 23.9% would first talk to another Ph.D. candidate, 23.9% to their daily supervisor, 20.7% to their doctoral advisor, 20.7% to the project leader, 7.6% to the confidential counselor, and 3.3% to someone else. The third category of Ph.D. candidates (15.5%) indicated they would first take a more *pragmatic approach* before doing anything else. They would only want to decide when, for example, more information is provided, new data is collected, or more analyses are conducted. The fourth category of Ph.D. candidates (10.5%) is afraid the situation *might backfire on them in a later stage of their career* which is their main argument for not proceeding with the paper, as is exemplified by this statement:

*“I’d rather finish my thesis later than put my career at risk.”*

The fifth category of Ph.D. candidates (8.7%) provides as the main argument that *they believe in* good scientific practice and a world where science serves to advance humanity:

*“Producing science and knowledge is part of academia so that humans can get closer to the ‘truth’, producing fake stuff is not part of academia and I don’t want to be part of that.”*

*“In the long-term, being honest provides the best answers to societal issues.”*

Lastly, we identified a group of Ph.D. candidates (8%) as “*at-risk.*” They either reported that if the pressure were high enough, they would proceed with the publication, as indicated by the following quote:

*“It’s not a solid yes, but a tentative one. I can image, just to be realistic, in terms of publishing pressures and not wanting to be out of contract, that this would be the best bet after all.”*

Or, they would follow their supervisor:

*“If the supervisors tell me it’s okay, I would try to publish the data.”*

Or, they simply have no qualms about it:

*“Since it will get me closer to obtaining my Ph.D.”*

The result of testing for manipulation effect was that for all scenarios, the null model, assuming no effect for condition, was preferred over the alternative model (all BF_01_’s < 1); see Supplementary Appendix C for detailed results.

### Intermediate Conclusion

The first study shows that at least some Ph.D. candidates are willing to publish results even if they know the data has been made up, the deletion of outliers is not adequately described, or if they are asked to split their papers into several sub-papers (i.e., salami-slicing). The percentage of Ph.D. candidates who actually experienced such a situation is low but not zero (see Table 2). Contrary to our expectations and although the manipulation checks were successful (see Supplementary Appendix B)–neither ethical leadership of the senior/supervisor nor transparency in the description resulted in differences in the Ph.D. candidates’ intended publishing behavior.

## Study 2–Expert Elicitation and Prior-Data Conflicts

The goal of Study 2 was to investigate how academic leaders believed Ph.D. candidates would respond to the three scenarios and to test whether the beliefs of the seniors about Ph.D. candidates’ behavior regarding QRPs conflicted with the observed data from Study 1.

### Methods

#### Participants and Design

We invited 36 academic leaders working at 10 different faculties of Social and Behavioral Sciences or Psychology in Netherlands–deans, vice-deans, heads of departments, research directors, and confidential counselors–to participate in the study and share what they believed Ph.D. candidates would answer when facing the three scenarios. The design of the study and how confidentiality would be ensured (i.e., personal characteristics would not be disclosed, answers would not be connected to specific data or results or used as predictors for explaining possible disagreements with the collected data in Study (2) was described in a face-to-face interview with the first author (RS). All academic leaders answered at least one scenario and very few skipped questions (response per scenario was 34, 35, and 33 from the 36 different leaders).

#### Analytic Strategy

The method used to obtain the necessary information from the experts is referred to as prior elicitation (O’Hagan et al., 2006), which is the process of extracting and creating a representation of an expert’s beliefs. During a face-to-face interview, we used the Trial-Roulette elicitation method to capture the beliefs of the seniors in a statistical distribution. This elicitation method was introduced by O’Hagan et al. (2006) and was validated by Johnson et al. (2010b); Veen et al. (2017), Zondervan-Zwijnenburg et al. (2017), and Lek and Van De Schoot (2018).

**FIGURE 1:**
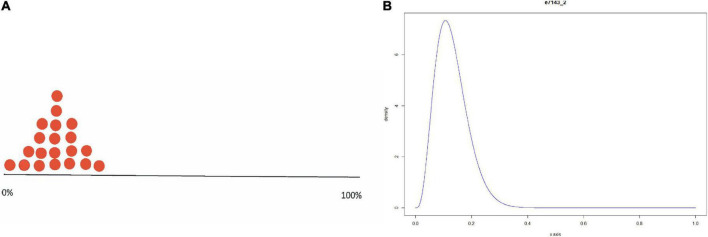
Example of a stickered distribution using **(A)** the trial roulette method and **(B)** the probability distribution obtained with the SHELF software (Oakley and O’Hagan, 2010).

**FIGURE 2:**
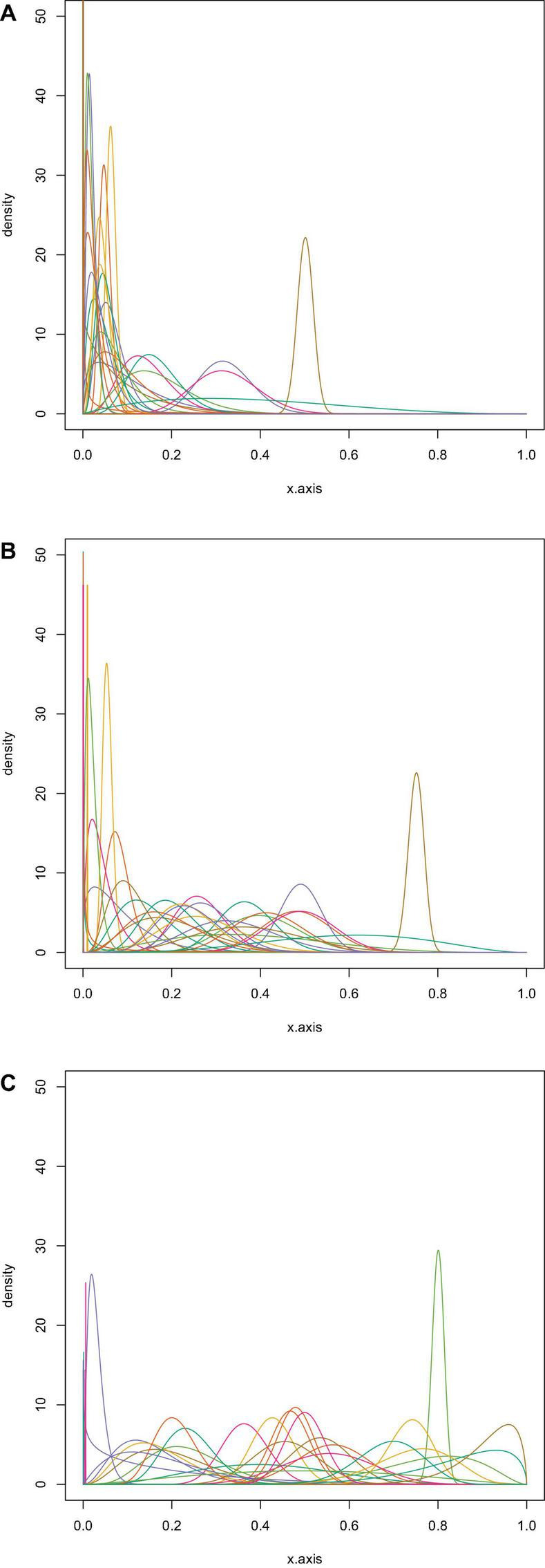
The parametric beta distributions based on the experts’ stickered distributions for Scenario 1 (**A**; *n* = 34), 2 (**B**; *n* = 35) and 3 (**C**; *n* = 33).

To obtain a proper representation of the experts’ beliefs about the percentage of Ph.D. candidates answering “yes” to the questions whether to publish the paper in the three scenarios, participants had to place twenty stickers, each representing five percent of a distribution, on an axis representing the percentage of Ph.D. candidates answering “yes” from 0% (left) to 100% (right). The placement of the first sticker at a specific position on the axis should indicate perceived likeliness by the expert for that value. In contrast, the other stickers represented uncertainty around this estimate, thereby creating a stickered distribution. The elicitation procedure resulted in one stickered distribution per expert per scenario, for a total of 102 valid distributions (six distributions could not be transformed into a parametric beta distribution). See Figure 1 for an example of such a stickered distribution and see Figure 2 for all the statistical distributions per scenario. The method we used to obtain statistical distributions based on the stickered distributions is published in van de Schoot et al. (2021b).

**FIGURE 3:**
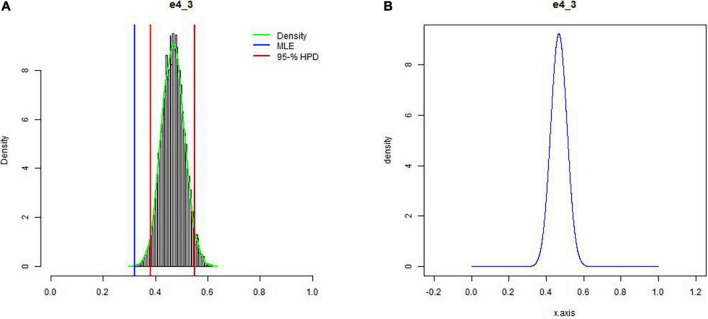
**(A)** A histogram of predicted data is shown based on the prior derived from the expert (shown in **B**). The red lines indicate the credibility interval of the prior predictive distribution, and the blue line the observed percentage. The probability value appeared to be <0.001, showing there is a prior-data conflict. A table with results per expert can be found on the OSF.

**FIGURE 4:**
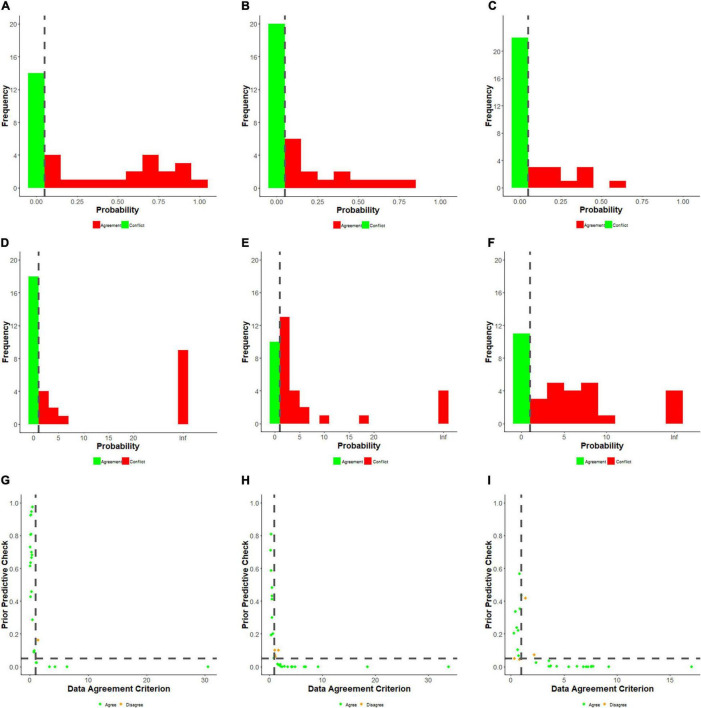
Results for the prior predictive check **(A–C)**, the DAC **(D–F)**, and for the combination of the two **(G–I)** for each scenario separately. The dotted line represents the cut-off values used. The green dots in **(G–I)**, indicate identical conclusions for both measures, and the orange dots indicate numerical differences. It should be noted all of these are boundary cases, for example, a PPC of 0.049 (conflict) and a DAC score of 0.98 (no conflict).

To examine whether the beliefs expressed by the senior academic leaders conflict with the observed data of the Ph.D. candidates (Study 1), we tested for an expert-data conflict. Box (1980) proposed using prior predictive distributions to test if the collected data was unlikely for this predictive distribution. Evans and Moshonov (2006) presented a variation, the prior-predictive check (PPC) computed per expert, and results in a value reflecting the existence of prior-data conflict. With the PPC, the prior distribution itself is used to predict various proportions that could have been observed. These predicted proportions can be used to assess the probability that the actual data proportion can be found using the prior distribution resulting in a probability value. When the value is less than 0.05, it reflects a prior-data conflict; see Figure 3. To cross-validate the results, we also computed the DAC developed by Bousquet (2008) and extended by Veen et al. (2018), where values >1 indicate a conflict. Since the results of both measures are highly comparable, see Figure 4; the results section below presents only the detailed PPC results. For a comparison between the two methods, see Lek and Van De Schoot (2019). The complete results, including annotated syntax, can be found on OSF (see text footnote 1).

### Results

As shown in van de Schoot et al. (2021b), 82% (40 and 18% for scenarios 2 and 3, respectively) of the academic leaders believed the percentage of Ph.D. candidates willing to publish a paper, even if they did not trust the data because of potential data fabrication, to be precisely zero (*n* = 8) or close to zero (*n* = 20).

When testing for prior-data conflicts for Scenario 1 (data fabrication), it appeared 20 experts (58.8%) showed no significant conflict with the data based on the PPC. Nine experts (26.5%) significantly underestimated the percentage of Ph.D. candidates willing to publish with fabricated data, while the remaining five (14.7%) overestimated this percentage. For Scenario 2 (Deleting Outliers), fewer experts (15; 42.9%) showed no significant conflict with the data. Only six experts (17.1%) significantly underestimated the percentage of Ph.D. candidates willing to publish with data that suppressed outliers, while 14 experts (40.0%) overestimated this percentage. For Scenario 3 (Salami Slicing), the lowest number of experts (11; 33.3%) showed no significant conflict with the data. Five experts (15.2%) significantly underestimated the percentage of Ph.D. candidates who would be willing to publish with data resulting from salami slicing, while most experts (17; 51.5%) overestimated this percentage.

### Intermediate Conclusion

Some academic leaders overestimated the percentage, and some were in tune with the outcomes of Study 1. However, academic leaders (too) often underestimate the willingness of Ph.D. candidates to “survive academia” utilizing fraudulent or QRPs. Underestimation is far more problematic because one student or researcher conducting QRPs can have profound implications. It is not easy to predict such behavior but expecting it to be non-existent, as several academic leaders believed, is overly optimistic. These findings indicate an awareness gap with senior academic leaders, a worrisome conclusion, given their position in the academic hierarchy and their role in policy development.

## Study 3–Vignette Study B

There were three goals for Study 3: First, to conceptually reproduce and extend the vignette study (we modified the description of the scenarios based on feedback to study 1, and we added three new scenarios). Our second goal was to investigate the influence of peer and elite pressure. The third goal was to examine honesty about having committed a QRP through the Bayes truth serum (Prelec, 2004).

### Methods

#### Participants, Procedure, and Design

For Study 3, we received a list of email addresses from one university of all Ph.D. candidates in two faculties (Psychology and Medicine), allowing us to send out our invitation email. We used the same online survey tool and set-up as study 1.

In total, 198 Ph.D. candidates completed the questions for at least one of the scenarios. The Ph.D. candidates were from two different faculties of one major university in Netherlands. Descriptive statistics on the sample can be found in Table 1.

#### Measures/Analytic Strategy

The first part of our survey was an adjusted version of the experiment applied in Study 1. Instead of three scenarios, we used only one scenario, an updated version of the Data Fabrication scenario adapted based on the Ph.D. candidates’ feedback in Study 1; see Supplementary Appendix A for the new text. The conditions for this experiment remained the same as in Study 1.

The second part of our experiment concerned the effect of varying levels of Peer and Elite pressure on participants’ publishing behavior when confronted with three QRPs: (1) Salami slicing (an adjusted version of the one used in Study 1), (2) gift authorship, i.e., adding an additional co-author who did not contribute to the article, and (3) leaving out relevant results. The effect of pressure was studied by adding vignettes that varied the pressure source (peer or elite) and the extent of pressure (low or high fictive percentages of the source of pressure partaking in QRPs). Again, we used Bayes Factors in JASP to test for the effects of the different conditions.

We also wanted to get a more accurate estimate of the prevalence of three QRPs (Salami Slicing, Gift Authorship, and Excluding Results) using the Bayesian truth serum (Prelec, 2004; John et al., 2012): a scoring algorithm that can be used to provide incentives for truthful responses. Participants were presented with an introductory text aimed at motivating participants to answer truthfully and asking them to answer three questions about the prevalence of each QRP in the department:

1. What percentage of your colleagues within your department has engaged in (QRP) on at least one occasion (on a scale from 0 to 100%)? (prevalence estimate).
2. Among those colleagues who have engaged in (QRP) on at least one occasion, what percentage would indicate that they have engaged in this research practice (on a scale from 0 to 100%)? (admission estimate).
3. Have you engaged in this research practice? (self-admission rate).

**FIGURE 5:**
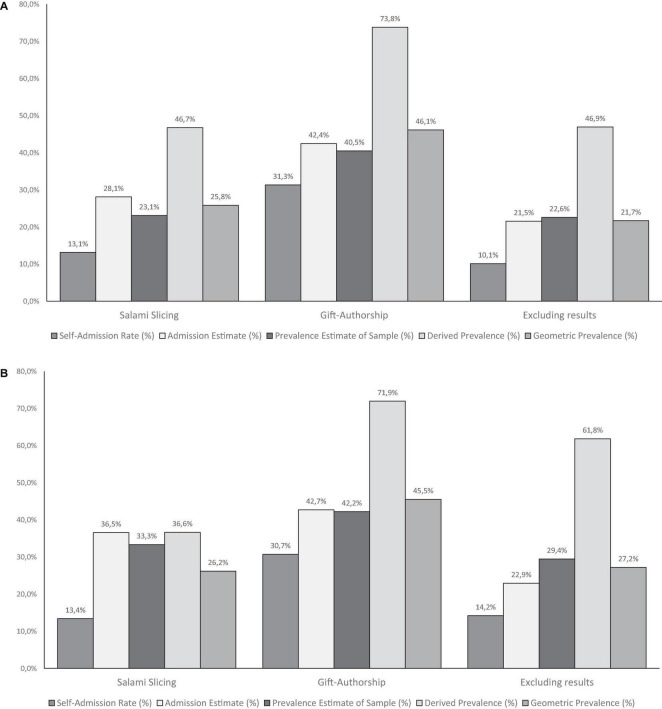
Bayesian truth serum Results of Study 3 **(A)** and Study 4 **(B)**.

Based on responses to the questions above, it is possible to compute a more realistic Actual Prevalence. John et al. (2012) suggested calculating the geometric mean of the self-admission rate, the average admission rate, and the prevalence estimate derived from the admission rate to come to a conservative Actual Prevalence Rate. The geometric mean is based on the product of the individual numbers (as opposed to the arithmetic mean, which is based on their sum); see Figure 5 and the OSF for annotated syntax (see text footnote 1).

### Results

#### Ethical Leadership and Transparency Experiment

Similar to Study 1, most Ph.D. candidates in the sample (92.4%) considered the data fabrication scenario fraudulent, but almost 10% would try to publish the results, and 5.5% reported experiencing such a situation. Again, the manipulation check was successful (see Supplementary Appendix B); the null model was always preferred over the alternative model (BF_01_ < 1). Also, again, the results indicate that the experimental conditions did not differ in publishing behavior; see Supplementary Appendix C for details.

#### Peer and Elite Pressure Experiment

Compared to Study 1, a much lower percentage of Ph.D. candidates considered the vignette of salami-slicing to be fraud (65.2 versus 16.6%). In contrast, the percentage of candidates who had been in such a situation doubled to 17%. The overall rates of participants who answered “yes, I would try to publish” were comparable to Study 1. The new scenarios of gift authorship and excluding information are considered fraud by more Ph.D. candidates. A majority of the Ph.D. candidates would publish the results in the scenario of gift authorship, but fewer had actually been in this situation, see Table 2. Concerning the *Peer and Elite Pressure* experiment, we did not find an effect for the experimental conditions (BFs < 1); see Supplementary Appendix C for detailed results. One exception was the model for salami slicing (Scenario 3r), which had a BF of 575, reflecting evidence in favor of a dependency in the contingency table. This result indicates higher pressure resulted in a higher percentage of Ph.D. candidates willing to publish the paper, especially when it concerned peer pressure.

#### Bayesian Truth Serum

Figure 5A shows our findings using the Bayesian truth serum. For example, 31% of the participants admitted to using the practice of gift authorship, much higher than for the other two scenarios. They expected that 40% of their colleagues did the same but that only 42% would admit doing so, leading to a Derived Prevalence Estimate of 73%. The conservative (geometric) prevalence rate would then be 46%, 14% more than the self-admission rate, comparable with the other two scenarios, 12, and 11%, respectively.

## Study 4–International Replication Study

The goal of the fourth study was to replicate the experiments of Study 3 and compute Bayes Factors for testing the replication effect of the Bayesian truth serum questions.

### Methods

#### Participants, Procedure, and Design

The Ph.D. candidates were from 3 Social Sciences faculties in Belgium. We applied an identical procedure to Study 3. In total, 127 Ph.D. candidates completed the questions for at least one scenario. Descriptive statistics on the sample can be found in Table 1.

#### Analytic Strategy

First, we computed a Bayes Factor similar to the Bayes Factor we used in the previous sections to test the manipulation check and experimental conditions (H_0_ = no effect). Second, we used the Equality of Effect Size Bayes Factor (Bayarri and Mayoral, 2002), which provides direct support, or lack thereof (i.e., H_0_), to whether the effect size found in the original study (Study 3) equals the effect size found in the replication attempt (Study 4). Third, we used the Bayes Factor Test for Replication Success (Verhagen and Wagenmakers, 2014) which is a test of the null hypothesis (H_0_ = no replication) versus the alternative replication hypothesis (successful replication, H_*rep*_). Annotated R-code to reproduce our results can be found on the OSF (see text footnote 1).

### Results

The overall percentage of participants who answered “yes, I would try to publish” is shown in Table 2.

For the *Supervisor and Data Transparency* experiment, as shown in Supplementary Appendix B, the manipulation check worked, but, as before, we did not find an effect for the experimental conditions; see Supplementary Appendix C for detailed results. These results mean that the experimental conditions did not result in differences in publishing behavior.

The Bayes truth serum results can be found in Figure 5B, and the percentages are very similar to those of Study 3. Table 3 displays the results of testing for a replication effect. For both studies and all three questions and scenarios, the Bayes Factors show extreme support of the percentages not being zero (see the results in the column titled BF1). The Bayes Factor for replication success (BF2) also shows great support for replicating the effects found in Study 3. The Equality of effect sizes Bayes Factor (BF3) provides support for some combinations, for example, the self-admission rate of the Salami slicing scenario with a BF of 13.74 and observed percentages of 13.13–13.39 [note that this Bayes Factor is typically much smaller (Verhagen and Wagenmakers, 2014)]. For some other conditions, there is less or even no support. In all, the percentages are pretty similar with similar effect sizes.

**TABLE 3:** Results of the Bayesian test of replication where Original refers to Study 3 and Replication refers to Study 4.

Question	Scenario	Study	Mean	SD	*t*	BF1	BF2	BF3
Admission estimate	Salami	Original	28.10	32.45	12.18	4.51E + 22		
		Replication	36.54	31.19	13.20	2.69E + 22	5.30E + 22	0.67
	Gift authorship	Original	42.45	35.67	16.75	2.74E + 36		
		Replication	42.69	32.07	15.00	4.59E + 26	6.71E + 27	6.67
	Excluding results	Original	21.54	29.81	10.16	4.99E + 16		
		Replication	22.93	26.81	9.64	6.94E + 13	6.79E + 14	7.10
Prevalence estimate	Salami	Original	23.07	27.69	11.72	1.91E + 21		
		Replication	33.31	30.39	12.35	2.45E + 20	7.87E + 20	1.38
	Gift authorship	Original	40.48	34.78	16.38	2.11E + 35		
		Replication	42.19	29.96	15.87	4.63E + 28	3.12E + 29	2.16
	Excluding results	Original	22.58	27.47	11.57	6.48E + 20		
		Replication	29.44	29.52	11.24	5.05E + 17	3.96E + 18	4.65

*BF1 refers to the Bayes Factor testing whether the estimate is zero or not. BF2 refers to the Bayes Factor Test for Replication Success. BF3 refers to the Equality of Effect Size Bayes Factor.*

## General Discussion

The scientific community is where early career researchers such as Ph.D. candidates are socialized and develop their future norms of scientific integrity. Although there are positive indications in the public debate that QRPs are no longer acceptable, our results show that an alarming percentage of Ph.D. candidates still reported intentions to conduct fraud when under pressure, even when asked about it in hypothetical scenarios where social desirability is probably quite prevalent. QRPs can be a sensitive topic that may lead to social desirability response bias or untruthful responses (consciously or unconsciously), possibly due to obedience to authority. We consider even one Ph.D. candidate reporting intentions to commit fraud an alarming number. The Bayesian truth serum results gave far higher scores than the survey vignettes and are meant to be more trustworthy. So, the qualitative data indicates that publication pressure (surviving in academia) and supervisors’ norms seem to drive the intention to conduct fraud.

Contrary to our expectations, and although the manipulation checks were all successful, neither ethical leadership of the senior/supervisor nor data transparency affected these vignettes on the Ph.D. candidates’ intended publishing behavior. More worrying, academic leaders–such as deans and heads of departments—might have a blind spot for the pressure Ph.D. candidates may experience to conduct QRPs or even fraud. Academic leaders do not always have an accurate, up-to-date perception of Ph.D. candidates’ willingness to engage in QRPs; eight leaders put all their density mass on exactly 0%, see Figure 5A in van de Schoot et al. (2021b). Some academic leaders in this study underestimated the inclination of Ph.D. candidates to conduct fraud or QRPs, although it must be said that some experts overestimated the percentage. It appeared not easy to predict such behavior but expecting it to be non-existent is overly optimistic.

All in all, the pressure to conduct QRPs or even commit fraud remains a significant problem for early-career scientists. We should keep an open eye for the possibility that early career researchers at least consider committing fraud when under pressure clears the way for discussing such practices. In this respect, it is imperative to inform senior academic leaders that their estimates of QRPs occurrence may be off. And although the awareness gap can go both ways in terms of over and underestimating the probability Ph.D. candidates would commit QRPs, it should be clear that underestimation could lead to more severe consequences in terms of scientific accuracy and rigor. Supervisors should take the initiative in having open discussions with the Ph.D. candidates in their department about good scientific practice versus unethical behavior. Leaders in general such as deans, vice-deans, heads of department, research directors, and confidential counselors should develop policies to address and prevent fraud and QRPs. It may seem an obvious statement to many academics. Still, as the responses in the current studies show, there are supervisors and academic leaders who do not think QRPs are a problem when they clearly still are.

The applied studies’ strengths lay not only in the use of innovative Bayesian methods, but external validity is also supported using surveys and open answer formats, interviews, an experiment, and conceptual replication. The analyses focused on the quantitative aspects of the data to demonstrate the Bayesian methods outlined in the aim of the manuscript. We have added a report on the OSF (see text footnote 1) for interested readers with the descriptive qualitative responses and frequencies.

Although we expect these results to be generalizable (as supported by the replication study), the sample from Belgium may share similarities to Netherlands samples. Generalization to other countries and cultures will, of course, benefit from additional research and further future replication. Another limitation is the lack of a baseline condition without fraudulent research practices. Future studies could include conditions or scenarios without QRPs for comparison purposes. We also did not evaluate potential differences in “trying to publish” between Ph.D. candidates who reported encountering such QRP scenarios and those who have not. Future research may benefit by designing a study to examine whether experiencing these situations results in fraud beliefs or publishing decisions versus hypothetical scenarios.

In sum, supervisors, deans, and other faculty must keep in mind that Ph.D. candidates can be under more pressure than they realize and might be susceptible to using QRP.

## Conclusion

More and more scientists have started to use Bayesian methods, and we encourage researchers to use the full potential of Bayesian methods. In this article, we demonstrated the application of some less commonly applied Bayesian methods by showcasing the use of expert elicitation, prior-data conflict tests, the Bayes truth serum, and testing for replication effects. As in all studies, many methodological and analytical decisions were made. While this could be seen as a limitation, we believe this is part of the transition toward Open Science. Therefore, to enable reproducibility, we shared all the underlying data and code following the FAIR principles: findability, accessibility, interoperability, and reusability. We hope our endeavor inspires other scientists to FAIR-ify their own work and provide the opportunity for other researchers to evaluate other alternative choices.

## Data Availability Statement

The datasets presented in this study can be found in online repositories. The names of the repository/repositories and accession number(s) can be found below: https://osf.io/raqsd/.

## Ethics Statement

The entire study was approved by the Ethics Committee of the Faculty of Social and Behavioral Sciences at Utrecht University (FETC15-108). The patients/participants provided their written informed consent to participate in this study.

## Author Contributions

RS, SG, and LT designed the study, developed the questionnaires, and coded the open answers for Study 1. SW was in charge of data collection and conducted most of the analyses of Study 1, 3, and 4 together with IA. RS collected the expert data for Study 2. EG was in charge of the elicitation procedure for Study 2 under supervision of RS and SW and conducted the analyses for Study 2 together with DV. EMG dealt with all changes and updates required for the revision. All authors contributed significantly to the writing process and preparation of the Supplementary Material.

## Conflict of Interest

The authors declare that the research was conducted in the absence of any commercial or financial relationships that could be construed as a potential conflict of interest.

## Publisher’s Note

All claims expressed in this article are solely those of the authors and do not necessarily represent those of their affiliated organizations, or those of the publisher, the editors and the reviewers. Any product that may be evaluated in this article, or claim that may be made by its manufacturer, is not guaranteed or endorsed by the publisher.

## Footnotes

1
https://osf.io/raqsd/
