# Supplementary material for: The Use of Questionable Research Practices to Survive in Academia Examined With Expert Elicitation, Prior-Data Conflicts, Bayes Factors for Replication Effects, and the Bayes Truth Serum
Source: Front Psychol. 2021 Nov 29;12:621547. doi: 10.3389/fpsyg.2021.621547 (PMC8667468; doi:10.3389/fpsyg.2021.621547)
Supplement: Supplementary file 1 [file Data_Sheet_1.docx]

Appendix A – Design of Studies

and Text of Vignettes

# study 1 – Vignette Study A

### **Text of Vignettes**

Table A.1 presents the text of the vignettes that was used for the PhD-candidates in Study 1 and for the seniors in Study 2.

| Table A.1. |
| --- |
| **Introduction**  Suppose you have been working on this research project with the project leader and senior team member for a few months. The following situation occurs. Together with the project leader and the senior team member you are developing an article. You are in charge of the data analysis. … |
| **Scenario 1 - data fabrication**:  While you are working on the analysis, you discover that something is wrong with the data: you have good reasons to assume the data has been made up, most likely by the senior team member, who was responsible for data collection. You discuss this point of concern with the senior team member, but you have not discussed this with the project leader. The senior team member advises you to use the data anyway because it leads to very interesting conclusions. You figure that publishing the results based on these data might result in a very good article which will be crucial in allowing you to finish your thesis in time. |
| **Scenario 2 - deleting outliers to get significant results:**  You checked the data and there appears to be no problem with it. However, your most important hypotheses are not supported by the data. You discuss this point of concern with the senior team member, but you have not discussed this with the project leader. The senior team member proposes to re-analyze the data together. Before doing so, he removes some outliers / interview quotes that, according to the senior member, disturb the data. He provides no further information. The new analysis shows support for your hypotheses. The senior team member advises you to use this data because it leads to very interesting conclusions. You figure that publishing the results based on this data might result in a very good article which will be crucial in allowing you to finish your thesis in time. |
| **Scenario 3 - salami slicing:**  You checked the data and there appears to be no problem with it. Your analysis shows support for your main hypotheses. However, with the current analysis you appear to be able to publish just one article based on this research project. You discuss this point of concern with the senior team member, but you have not discussed this with the project leader. The senior team member asks you to analyze the data in such a way that the group can publish three similar articles instead of one, based on the same dataset. The three proposed articles will differ from each other only marginally. You figure that publishing three articles instead of one will be crucial in allowing you to finish your thesis in time. |
| **Question:**  would you try to publish the results of this study? |

### **Research transparency items used**

**Leadership and transparency**

In this part, we would like to ask you some questions about how you would assess the project leader in the presented situations.

**Please answer the following statements about the project leader.**

**The project leader...**

Please choose the appropriate response for each item:

|  | **Strongly disagree** | **Disagree** | **Neutral** | **Agree** | **Fully agree** | **N.A./don't know** |
| --- | --- | --- | --- | --- | --- | --- |
| **…shows a strong concern for ethical and moral values** |  |  |  |  |  |  |
| **…communicates clear ethical standards for team members** |  |  |  |  |  |  |
| **…keeps his actions consistent with his stated values (“walks the talk”)** |  |  |  |  |  |  |
| **…opposes the use of unethical practices to increase performance** |  |  |  |  |  |  |
| **…holds team members accountable for using ethical practices in their work** |  |  |  |  |  |  |

**Please answer the following statements about the project data in the presented situations.**

**The research material in the project...**

Please choose the appropriate response for each item:

|  | **Strongly disagree** | **Disagree** | **Neutral** | **Agree** | **Fully agree** | **N.A./don't know** |
| --- | --- | --- | --- | --- | --- | --- |
| **…is likely to be monitored by other researchers** |  |  |  |  |  |  |
| **…will be accessible to the other scholars in the near future** |  |  |  |  |  |  |
| **…are visible to scholars outside the project** |  |  |  |  |  |  |
| **…are dealt with in a very transparent manner** |  |  |  |  |  |  |

**Participants Study 1**

The PhD candidates for Study 1 were recruited from ten Social Sciences or Psychology faculties at eight universities in the Netherlands out of ten major universities that have social science or psychology faculties.

| Table A.2. The invitation text used in Study 1. |
| --- |
| **Invitation letter:**  First, we would like to ask some questions about where you work and what your major field of study is. We will only use this to relate to your answers at an aggregate level. This information can never be tracked down to you personally. The survey takes 15 to 20 minutes to complete and will be handled confidentially. Please read below the Note on Privacy to see how we safeguard the confidentiality of results. Note: If you have specific questions or concerns about research fraud, we advise you to contact your local supervisor or confidential counsellor. It is important to know that this survey is completely anonymous. We won’t be able to link your responses back to your e-mail address or to you personally. The survey software makes sure all identifying information is separated from the survey responses. |
| **Furthermore, on the introduction page of the survey, we included the following:**  This survey is anonymous. The record of your survey responses does not contain any identifying information about you, unless a specific survey question explicitly asked for it. If you used an identifying token to access this survey, please rest assured that this token will not be stored together with your responses. It is managed in a separate database and will only be updated to indicate whether you did (or did not) complete this survey. In no way shall identification tokens be matched with survey responses. |

### **Experiment Study 1**

Table A.3 below preceding the vignettes of Table A.1. The top row contains the text to describe leadership, while the bottom row illustrates the information we provided on the data.

| Table A.3. Experimental conditions of Study 1. | | |
| --- | --- | --- |
|  | **Ethical and open** | **Non-ethical and closed** |
| **Leadership** | The project leader is a famous scholar. He emphasizes that research ethics are crucial for both himself and his team. During various meetings you discussed ethical dilemmas that may occur when doing research, and he provided suggestions on how you and the team could deal with such issues. Furthermore, he takes strict action against people who violate ethical principles. For instance, a few years ago he fired a senior researcher working under him who was found guilty of plagiarism. | The project leader is a famous scholar. During various project meetings he provided no opportunity to discuss ethical dilemmas that may occur when doing research, nor did he provide suggestions about how you and the team could deal with these issues. Furthermore, he has never taken any action against people who have violated ethical principles. For instance, a few years ago a senior researcher working under him was found guilty of plagiarism, but no actions were taken to prevent such situations from happening again. |
| **Data** | The project is funded by a grant. When accepting the grant, the project leader and the senior team member agreed with the funding agency that all materials and raw research data will be disclosed on a website to make it available for other scholars, who can check your work and use the data for their own research. | The project is funded by a grant. When accepting the grant, the project leader and the senior team member agreed with the funding agency that you can keep all materials and raw research data to yourself and that there is no obligation whatsoever to disclose these materials to other researchers (such as on a website or online repository). |

# study 3 – Vignette Study B

| Table A.4. Text of the vignettes as presented to the PhD student as part of the first experiment of Study 3. | |
| --- | --- |
| **Introduction**  Now we would like you to read the hypothetical situation below about a research project. Try to really imagine yourself working on this particular research project. Reflect on what you think of the project and how you would behave. Note that there are no ‘right’ or ‘wrong’ answers. Remember that the survey is completely anonymous. … | |
| **Scenario 1 - data fabrication**:  You need one more publication to finish your dissertation in time. You have been working on an article based on the data of an associate professor at your department. He is not one of your dissertation supervisors, but you do work together because he collected the research material and he wants to be co-author of this article.  After 6 months of writing and rewriting, the paper is nearly finished and ready to be submitted. You and the associate professor figure that the results and conclusions in this article are groundbreaking and could be published in a top journal in your field.  You did most of the writing and the associate professor did most of the analysis because it was his data. You decide – just to be sure – to have a final look at the data. When comparing the raw data with the analysis it seems that for no clear reason some cases/relevant interview quotes have been left out. The unadjusted data leads to different conclusions that are unpublishable.  Because you spent the last 6 months on this paper and because you need this article to finish in time, you ask the associate professor for an explanation. He cannot give a proper explanation and tells you that this is how science works in practice.  The next day you have a meeting with your dissertation supervisor. Here, you address this issue. | |
| **Four conditions**:  Your supervisor says that you should not make life too difficult for yourself, while emphasizing the importance of publishing in top journals. Your supervisor leaves it up to you whether or not to submit the paper to the top journal.  Before you decide to submit, you look at the author instruction on the website of the journal. On the website, it is stated that prospective authors are obliged to submit all research materials, such as survey and/or interview data, along with the paper. This means that you have to disclose your research materials. | Your supervisor says that this is an unfortunate situation and condemns the behavior of the associate professor, while emphasizing the importance of good research practices. Your supervisor leaves it up to you whether or not to submit the paper to the top journal.  Before you decide to submit, you look at the author instruction on the website of the journal. On the website, there are no statements regarding submitting research materials (such as survey and/or interview data) along with the paper. Hence, you do not have to submit your research materials. |
| **Question:**  Would you try to publish the results of this study? | |

For the second part of Study 3, we compared responses of the PhD students across four conditions, which were combinations of two two-level factors, Percentage and Seniority:

1. High percentage and full professors
2. Low percentage and full professors
3. High percentage and PhD students
4. Low percentage and PhD students

The percentages reflected the proportions of full professors/PhD students at a different, imaginary, Dutch university who would pursue each of the three Questionable Research Practices.

| Table A.5. Text of the vignettes as presented to the PhD students as part of the second experiment of Study 3. |
| --- |
| **Introduction**  In the beginning of this year, we carried out a very similar study about research integrity at a different Dutch university. Below we will present to you some research situations we showed to them. We will share their response and are interested in how you would react. We have three situations we would like you to consider… |
| **Scenario 1: Salami slicing**  Together with a colleague you are working on an article. You are in charge of the data analysis. You checked the data and there appears to be no problem with it. Your analysis shows support for your main hypotheses. With the current analysis it seems that you will be able to publish one article based on this research project. Your colleague asks you to analyze the data in such a way that the group can publish three similar articles instead of one, based on the same dataset. The three proposed articles will differ from each other only marginally. You figure that publishing three articles instead of one will be of great value for your future career. Our results from the other Dutch university indicate that XX percent of XX would pursue publication of three articles instead of one. We have another situation we would like you to consider. This situation is unrelated to the previous situation. |
| **Four conditions**:  86 percent of the full professors  19 percent of the full professors  86 percent of the PhD students  19 percent of the PhD students |
| **Scenario 2: Gift authorship**  You are working with a colleague on an article. You and your colleague figure that the results and conclusions in this article are groundbreaking and could be publishable in a top journal in your field. You and the co-author both wrote equal parts of the paper. However, when you check the final paper before submission you see that a full professor has been added as a third author to the paper. He did not provide a substantial contribution to the paper and you wonder whether he truly understands the paper and could publicly defend it. When you ask your colleague he tells you he added the professor because he is a very prominent scholar in the field. Publishing with this person almost certainly increases the number of citations and more general use of the article. This can enhance your career chances. Our results from the other Dutch University indicate that XX percent of XX would publish the article, including the new author. We have one final situation we would like you to consider. This situation is unrelated to the previous situation. |
| **Four conditions**:  91 percent of the full professors  23 percent of the full professors  91 percent of the PhD students  23 percent of the PhD students |
| **Scenario 3: Excluding Results**  You and your colleague figure that the results and conclusions in the article you are working on are groundbreaking and could be publishable in a top journal in your field.  Just before submitting the paper you decide to check the data analysis your colleague carried out. You discover that – although the analyses are correct – not all information has been reported in the results section. Reporting this information would force you to tone down the conclusions of your paper, making it highly unlikely that it is going to be publishable in the top journal you were considering. Our results from the other Dutch University indicate that XX percent of XX would pursue publication of this article without reporting this information. |
| **Four conditions**:  78 percent of the full professors  12 percent of the full professors  78 percent of the PhD students  12 percent of the PhD students |
| **Question:**  would you try to publish the results of this study? |

Bayes Truth Serum Questions

1. What percentage of your colleagues within your department has engaged in [QRP] on at least one occasion (on a scale from 0 to 100%)? **prevalence estimate**

### Among those colleagues who have engaged in [QRP] on at least one occasion, what percentage would indicate that they have engaged in this research practice (on a scale from 0 to 100%)? **admission estimate**

1. Have you engaged in this research practice? **self-admission rate**

To motivate participants to answer truthfully to each question, the QRP scenarios were preceded by the following message:

“*These three pieces of information will help to develop more accurate estimates of the prevalence of each practice. Furthermore, this information will let us apply a formula called the Bayesian Truth Serum. Since this survey is anonymous, we cannot compensate you for your time and effort. Instead, we will make a donation to a charity of your choice. The size of this donation will be determined by the truthfulness of your answers about the three research practices, as assessed by the Bayesian Truth Serum. The important property of the formula is that it rewards truthful answers. This means that truthful answers about your practices will increase the donation made on your behalf (and will also tend to increase the donations made on behalf of other respondents). For the purpose of this survey, it is not necessary for you to understand how the formula works, although the theoretical paper from Science, which includes a short abstract, is available here.”*
