# Supplementary material for: The Use of Questionable Research Practices to Survive in Academia Examined With Expert Elicitation, Prior-Data Conflicts, Bayes Factors for Replication Effects, and the Bayes Truth Serum
Source: Front Psychol. 2021 Nov 29;12:621547. doi: 10.3389/fpsyg.2021.621547 (PMC8667468; doi:10.3389/fpsyg.2021.621547)
Supplement: Supplementary file 2 [file Data_Sheet_2.docx]

Appendix B - Manipulation checks

# study 1

We executed two Bayesian ANOVAs (BANOVAs) in JASP to test whether the experimental manipulation had worked. In the first BANOVA, we examined whether the High vs. Low Ethics conditions could be differentiated by the participants’ mean score on the Ethical Leadership Scale. A model including the Ethics condition was preferred over the null model (BF = 1.38E+47), indicating that there was a mean difference in Ethical Leadership between the two conditions. As expected, participants in the High Ethics condition had, on average, a higher score on Ethical Leadership than participants in the Low Ethics condition. See also the means and credibility intervals in Table B.1.

In the second BANOVA, we examined whether the Transparent vs. Not Transparent conditions could be differentiated by the participants’ mean score on the Data Transparency Scale. A model including the Transparency condition was preferred over the null model (BF = 1.25E+20), indicating that there was a mean difference in Data Transparency between the two conditions. As expected, participants in the Not Transparent condition had, on average, a lower score on Data Transparency than participants in the Transparent condition (table B.1).

*Table B.1. Means and credibility intervals for the four conditions in Study 1 for Ethical leadership and Transparency*

|  | Project leader | | Project data | |
| --- | --- | --- | --- | --- |
|  | Non-ethical | Ethical | Non-transparent | Transparent |
| M | 1.98 | 3.57 | 2.19 | 3.23 |
| 95% CI | 1.88-2.09 | 3.42-3.71 | 2.07-2.32 | 3.09-3.38 |

# study 3

We executed two BANOVAs to test whether the experimental manipulation had worked. In the first BANOVA, we examined whether the High vs. Low Ethics conditions could be differentiated by the participants’ mean score on the Ethical Leadership Scale. The BF for a model including the Ethics condition was not preferred over the null model (BF = 1.23), and we refrain from drawing conclusions about a potential difference in Ethical Leadership between the two conditions, see also table B.2.

In the second BANOVA, we examined whether the Transparent vs. Not Transparent conditions could be differentiated by the participants’ mean score on the Data Transparency Scale. A model including the Transparency condition was *not* preferred over the null model (BF = 0.21), indicating that there was more support for no mean difference in Data Transparency between the two conditions. However, participants in the Not Transparent condition had, on average, only a slightly lower score on Data Transparency than participants in the Transparent condition (table B.2). The 95% CI for each group’s mean showed overlap, further indicating that the Transparency condition did not affect participants’ perceptions of data transparency.

*Table B.2. Means and credibility intervals for the four conditions in Study 3 for Ethical leadership and Transparency*

|  | Project leader | | Project data | |
| --- | --- | --- | --- | --- |
|  | Non-ethical | Ethical | Non-transparent | Transparent |
| M | 2.88 | 3.23 | 2.84 | 2.96 |
| 95% CI | 2.65-3.11 | 2.99-3.47 | 2.60-3.06 | 2.75-3.16 |

# study 4

We executed two BANOVAs to test whether the experimental manipulation had worked. In the first BANOVA, we examined whether the High vs. Low Ethics conditions could be differentiated by the participants’ mean score on the Ethical Leadership Scale. A model including the Ethics condition was slightly preferred over the null model (BF = 4.58), indicating that there was a small mean difference in Ethical Leadership between the two conditions. As expected, participants in the High Ethics condition have, on average, a higher score on Ethical Leadership than participants in the Low Ethics condition (table B.3).

In the second BANOVA, we examined whether the Transparent vs. Not Transparent conditions could be differentiated by the participants’ mean score on the Data Transparency Scale. A model including the Transparency condition was preferred over the null model (BF = 33.37), indicating that there was a mean difference in Data Transparency between the two conditions. Participants in the Not Transparent condition have, on average, a lower score on Data Transparency than participants in the Transparent condition (table B.3).

*Table B.3. Means and credibility intervals for the four conditions in Study 4 for Ethical leadership and Transparency*

|  | Project leader | | Project data | |
| --- | --- | --- | --- | --- |
|  | Non-ethical | Ethical | Non-transparent | Transparent |
| M | 2.65 | 3.16 | 2.48 | 3.13 |
| 95% CI | 2.379-2.913 | 2.886-3.432 | 2.217-2.744 | 2.586-3.397 |
