# Supplementary material for: The Use of Questionable Research Practices to Survive in Academia Examined With Expert Elicitation, Prior-Data Conflicts, Bayes Factors for Replication Effects, and the Bayes Truth Serum
Source: Front Psychol. 2021 Nov 29;12:621547. doi: 10.3389/fpsyg.2021.621547 (PMC8667468; doi:10.3389/fpsyg.2021.621547)
Supplement: Supplementary file 3 [file Data_Sheet_3.docx]

Appendix C - Detailed results experimental conditions

# study 1

We used JASP for computing Bayes Factors for contingency tables to examine whether the experimental conditions had an effect on the participants’ attitude towards publishing data or analyses that might have fallen victim to QRPs. The result was that for all scenarios the null model was preferred over the alternative model (BFs < 1). This means that the experimental conditions did not result in differences in publishing behavior. See Table C.1 for percentages of participants answering “yes” for each scenario, and Table C.2 and 3.3 for the Bayes Factors for the alternative models and the null model respectively. The overall percentages of participants who answered “yes, I would try to publish” were 5.9%, 12.3%, and 32.0% respectively.

*Table C.1. Percentage of participants who answered “yes, I would try to publish” for each scenario, for each condition.*

| **Ethical** | **Transparent** | **Scenario 1** | **Scenario 2** | **Scenario 3** |
| --- | --- | --- | --- | --- |
| Low Ethics | Not Transparent | 4.55 | 14.55 | 27.27 |
|  | Transparent | 3.57 | 9.82 | 32.14 |
| High Ethics | Not Transparent | 4.59 | 9.17 | 23.85 |
|  | Transparent | 11.01 | 11.93 | 32.11 |

Note. Scenario 1 N = 440, Scenario 2 N = 407, Scenario 3 N = 397.

*Table C.2. Bayes Factors for the alternative model for each scenario*

| **Model** | **Scenario 1** | **Scenario 2** | **Scenario 3** |
| --- | --- | --- | --- |
| Ethics x Transparency | 6.54e-3 | 2.20e-3 | 0.02 |

# study 3

## Main Experiment: Supervisor & Data Transparency

We used Bayesian contingency tables to examine whether the experimental conditions had an effect on the participants’ attitude towards publishing data or analyses that might have fallen victim to QRPs. For the included scenario the null model was preferred over the alternative model (BFs < 1). This means that the experimental conditions did not result in differences in publishing behavior. See Table C.3 for percentages of participants answering “yes” for each scenario, and Table C.4 for the Bayes Factors for the alternative model. The overall percentage of participants who answered “yes, I would try to publish” was 9.6%.

## Main Experiment: Peer and Expert Pressure

We used Bayesian contingency tables to examine whether the experimental conditions had an effect on the participants’ attitude towards publishing data or analyses that might have fallen victim to QRPs. For scenario four and five the null model was preferred over the alternative model (BFs < 1). For scenario 3r (salami slicing) the alternative model is preferred (BF = 575.01).
This means that, except for scenario 3r, the experimental conditions did not result in differences in publishing behavior. See Table C.5 for percentages of participants answering “yes” for each scenario, and Table C.6 for the Bayes Factors for the alternative models. The overall percentages of participants who answered “yes, I would try to publish” were 38.9%, 59.2%, and 12.1% respectively.

*Table C.3. Percentage of participants who answered “yes, I would try to publish” for each condition.*

| **Ethical** | **Transparent** | **Scenario 1r** |
| --- | --- | --- |
| Low Ethics | Not Transparent | 12.00 |
|  | Transparent | 7.69 |
| High Ethics | Not Transparent | 10.20 |
|  | Transparent | 8.51 |

Note. Scenario 1r N = 198. ‘r’=revised

*Table C.4. Bayes Factors for the alternative model*

| **Model** | **Scenario 1r** |
| --- | --- |
| Ethics x Transparency | 0.09 |

Note. ‘r’=revised

*Table C.5. Percentage of participants who answered “yes, I would try to publish” for each scenario, for each condition.*

| **Level** | **Source** | **Scenario 3r** | **Scenario 4** | **Scenario 5** |
| --- | --- | --- | --- | --- |
| Low Pressure | Peer | 24.14 | 46.55 | 10.34 |
|  | Expert | 18.52 | 40.74 | 9.26 |
| High Pressure | Peer | 58.33 | 58.33 | 14.58 |
|  | Expert | 35.71 | 57.14 | 7.14 |

Note. Scenario 1 N = 185, Scenario 2 N = 184, Scenario 3 N = 182. ‘r’=revised

*Table C.6. Bayes Factors for the alternative model for each scenario*

| **Model** | **Scenario 3r** | **Scenario 4** | **Scenario 5** |
| --- | --- | --- | --- |
| Pressure x Source | 575.01 | 0.21 | 7.01e-3 |

Note. ‘r’=revised

# study 4

## Main Experiment: Supervisor & Data Transparency

We used Bayesian contingency tables to examine whether the experimental conditions had an effect on the participants’ attitude towards publishing data or analyses that might have fallen victim to QRPs. For the included scenario the alternative model was slightly preferred over the alternative model (BFs > 1). However, the BFs are < 3, indicating only anecdotal evidence in favor of a dependency in the contingency table. See Table C.7 for percentages of participants answering “yes” for each scenario, and Table C.8 for the Bayes Factors for the alternative model. The overall percentage of participants who answered “yes, I would try to publish” was 13.4%.

## Main Experiment: Peer and Expert Pressure

We used Bayesian contingency tables to examine whether the experimental conditions had an effect on the participants’ attitude towards publishing data or analyses that might have fallen victim to QRPs. In short, for all scenarios the null model was preferred over the alternative model (BF s< 1). This means that the experimental conditions did not result in differences in publishing behavior. See Table C.9 for percentages of participants answering “yes” for each scenario, and Table C.10 for the Bayes Factors for the alternative models and the null model respectively. The overall percentages of participants who answered “yes, I would try to publish” were 32.8%, 58.8%, and 16.1% respectively.

*Table C.7. Percentage of participants who answered “yes, I would try to publish” for each condition.*

| **Ethical** | **Transparent** | **Scenario 1r** |
| --- | --- | --- |
| Low Ethics | Not Transparent | 23.53 |
|  | Transparent | 8.33 |
| High Ethics | Not Transparent | 13.79 |
|  | Transparent | 7.14 |

Note. Scenario 2 N = 127. ‘r’=revised

*Table C.8. Bayes Factors for the alternative model*

| **Model** | **Scenario 1r** |
| --- | --- |
| Ethics x Transparency | 0.61 |

*Table C.9. Percentage of participants who answered “yes, I would try to publish” for each scenario, for each condition.*

| **Level** | **Source** | **Scenario 3r** | **Scenario 4** | **Scenario 5** |
| --- | --- | --- | --- | --- |
| Low Pressure | Peer | 23.68 | 44.74 | 7.89 |
|  | Expert | 24.32 | 51.35 | 10.81 |
| High Pressure | Peer | 38.24 | 55.88 | 26.47 |
|  | Expert | 29.63 | 51.85 | 11.11 |

Note. Scenario 3r N = 118, Scenario 4 N = 118, Scenario 5 N = 117. ‘r’=revised

*Table C.10. Bayes Factors for the alternative model for each scenario*

| **Model** | **Scenario 3r** | **Scenario 4** | **Scenario 5** |
| --- | --- | --- | --- |
| Pressure x Source | 0.1 | 0.06 | 0.85 |

Note. ‘r’=revised
